# Molecular Chaperones and miRNAs in Epilepsy: Pathogenic Implications and Therapeutic Prospects

**DOI:** 10.3390/ijms22168601

**Published:** 2021-08-10

**Authors:** Leila Zummo, Alessandra Maria Vitale, Celeste Caruso Bavisotto, Marco De Curtis, Rita Garbelli, Anna Teresa Giallonardo, Carlo Di Bonaventura, Martina Fanella, Everly Conway de Macario, Francesco Cappello, Alberto J. L. Macario, Antonella Marino Gammazza

**Affiliations:** 1 Department of Biomedicine, Neuroscience and Advanced Diagnostics, Section of Human Anatomy, University of Palermo, 90127 Palermo, Italy; leilazummo@yahoo.it (L.Z.); alessandramaria.vitale@unipa.it (A.M.V.); celeste.carusobavisotto@unipa.it (C.C.B.); francesco.cappello@unipa.it (F.C.); 2 Department of Neurology and Stroke Unit, A.R.N.A.S. Ospedale Civico—Di Cristina Benfratelli, 90127 Palermo, Italy; 3 Euro-Mediterranean Institute of Science and Technology (IEMEST), 90139 Palermo, Italy; AJLMacario@som.umaryland.edu; 4 Epilepsy Unit, Fondazione IRCCS Istituto Neurologico Carlo Besta, 20133 Milan, Italy; marco.decurtis@istituto-besta.it (M.D.C.); rita.garbelli@istituto-besta.it (R.G.); 5 Department of Human Neurosciences “Sapienza”, University of Rome, 00185 Rome, Italy; annateresa.giallonardo@uniroma1.it (A.T.G.); carlo.dibonaventura@uniroma1.it (C.D.B.); fanella.martina@gmail.com (M.F.); 6 Policlinico Umberto I, 00161 Rome, Italy; 7 Department of Microbiology and Immunology, School of Medicine, University of Maryland at Baltimore-Institute of Marine and Environmental Technology (IMET), Baltimore, MD 21202, USA; econwaydemacario@som.umaryland.edu

**Keywords:** epilepsy, temporal lobe epilepsy, miRNA, chaperone system, molecular chaperones

## Abstract

Epilepsy is a pathologic condition with high prevalence and devastating consequences for the patient and its entourage. Means for accurate diagnosis of type, patient monitoring for predicting seizures and follow up, and efficacious treatment are desperately needed. To improve this adverse outcome, miRNAs and the chaperone system (CS) are promising targets to understand pathogenic mechanisms and for developing theranostics applications. miRNAs implicated in conditions known or suspected to favor seizures such as neuroinflammation, to promote epileptic tolerance and neuronal survival, to regulate seizures, and others showing variations in expression levels related to seizures are promising candidates as useful biomarkers for diagnosis and patient monitoring, and as targets for developing novel therapies. Components of the CS are also promising as biomarkers and as therapeutic targets, since they participate in epileptogenic pathways and in cytoprotective mechanisms in various epileptogenic brain areas, even if what they do and how is not yet clear. The data in this review should help in the identification of molecular targets among the discussed miRNAs and CS components for research aiming at understanding epileptogenic mechanisms and, subsequently, develop means for predicting/preventing seizures and treating the disease.

## 1. Introduction

In the last decade or so, there has been an impressive progress in many fields of medicine, including the neurosciences, stemming from various developments such as novel molecular biologic methods, genomics, proteomics, identification and characterization of miRNAs, and definition of the chaperone system (CS) and its diseases. A better understanding of miRNAs and the CS has had a significant impact in neurology due to their roles in the development, maturation, and pathology of the central and peripheral nervous systems. Here, we will briefly discuss some of the advances pertinent to miRNAs and the CS in epilepsy.

### 1.1. Epilepsy: A Spectrum of Disorders

Epilepsy is one of the most common and serious neurological disorders, affecting nearly 50 million people worldwide and resulting in a major socioeconomic burden [1,2] (WHO report: https://www.who.int/news-room/fact-sheets/detail/epilepsy accessed on 6 August 2021). It is a chronic neurological disorder characterized by a predisposition to generate recurrent unprovoked epileptic seizures. A seizure is “a transient occurrence of signs and/or symptoms due to abnormal excessive or synchronous neuronal activity in the brain”, as defined by the International League Against Epilepsy (ILAE, [3,4]). Seizures are usually described as having focal or generalized onset; however, in certain cases, the onset cannot be readily determined (unknown onset). Focal onset seizures are generated by abnormal activity starting from a brain region, and they are further classified “with intact consciousness” and “with altered level of consciousness”, whereas generalized seizures arise in bilaterally distributed brain networks [5,6]. Another definition states: epilepsy is a “chronic disease of the brain characterized by an enduring (i.e., persisting) predisposition to generate seizures, unprovoked by any immediate central nervous system insult, and by the neurobiologic, cognitive, psychological, and social consequences of seizure recurrences” [7].

Epilepsy may not be considered a single condition but a spectrum of disorders that share an alteration in the brain that increases the likelihood of seizures.

The etiologies of epilepsy and epileptic syndromes are diverse, for instance brain lesions, neoplasms, malformation of cortical development, and tuberous sclerosis complex are major causes of drug-resistant epilepsy and sometimes require surgical interventions [8]. When the etiology is known, epilepsy is categorized as symptomatic or of structural/metabolic etiology, and if the etiology is unknown, the disorder is classified as cryptogenic [4]. The course of epileptic syndromes and their response to the available therapies differ. Seizures can be controlled with appropriate drugs in approximately 70% of cases, but the remaining 30% of patients are intractable with standard medical treatments [9,10]. Hence, there is an urgent need for the clarification of mechanisms of epileptogenesis and for the development of antiepileptogenic therapies [11].

### 1.2. Temporal Lobe Epilepsy (TLE)

Temporal lobe epilepsy (TLE) is the most common form of focal epilepsy in adults, accounting for 60% of cases [12]. Several subclassifications exist regarding the neuroanatomical origin of the seizures, with the distinction between mesial and lateral temporal seizure onsets being one of the most widely used.

Mesial temporal lobe epilepsy (MTLE) arises in the hippocampus, parahippocampal gyrus, and amygdale, while lateral temporal lobe epilepsy (LTLE) arises in the neocortex on the outer surface of the temporal lobe of the brain.

More than 90% of patients with MTLE report a visceral aura, most commonly an epigastric sensation with a tendency to increase with time. Other auras consist of an abnormal sense of taste, an aversive smell, déjà vu, or a dreamy sensation. Fear is the most reported affective symptom, although other complex emotional symptoms may also occur. Autonomic symptoms include changes in skin color, blood pressure, heart rate, and piloerection. As seizures usually involve areas of the limbic system, which control emotions and memory, some individuals may have memory problems [13,14].

The most common pathology underlying mesial temporal lobe epilepsy is hippocampal sclerosis (HS). It is characterized by a severe loss of the principal neurons associated with the widening of the granule cell layer of the dentate gyrus, termed granule cell dispersion, which is observed in about 40–50% of surgical temporal lobe specimens [15,16]. This condition is often associated with a history of febrile seizures in infancy. Temporal lobe epilepsy associated with HS is often progressive with the worsening of seizures, impairment of cognitive function, and psychiatric disorders, and is accompanied by significant morbidity and mortality [17,18].

Mesial temporal lobe epilepsy, especially with pathologically or radiologically proven mesial temporal sclerosis, is considered a highly refractory and drug-resistant type of epilepsy. Pharmaco-resistance is the “failure of an adequate trial of two tolerated, appropriately chosen, and used antiepileptic drug (AED) schedules (whether as monotherapies or in combination) to achieve sustained seizure freedom” as per ILAE [8]. In this scenario, resection surgery has been recognized as an effective treatment for pharmaco-resistant TLE-HS [19,20,21,22]. However, one third of surgically treated patients presented unfavorable results. Considering the variety of treatment options that are available, future efforts should focus on advocating for early referral of patients with drug-resistant TLE-HS for more comprehensive epilepsy management.

It is clear from current knowledge that pharmaco-resistance in epilepsy is a multifactorial phenomenon, but, based on existing proofs, more work is needed to test, reinforce, and integrate the current theories, with the goal of guiding the development of better epilepsy therapies. Until more data become available, it is fair to say that transporter overexpression is most likely not the only factor that plays in pharmaco-resistance, and that the best evidence available only supports the plausibility for the clinical role of efflux transporters in refractory epilepsy. Obviously, a better understanding of the relationship between epileptogenesis and the development of pharmaco-resistance will help to treat epilepsy by preventing the development of pharmaco-resistance against antiepileptic drugs.

## 2. MiRNAs: Localization, Functions, and Roles during Neurodegeneration

**Figure 1:**
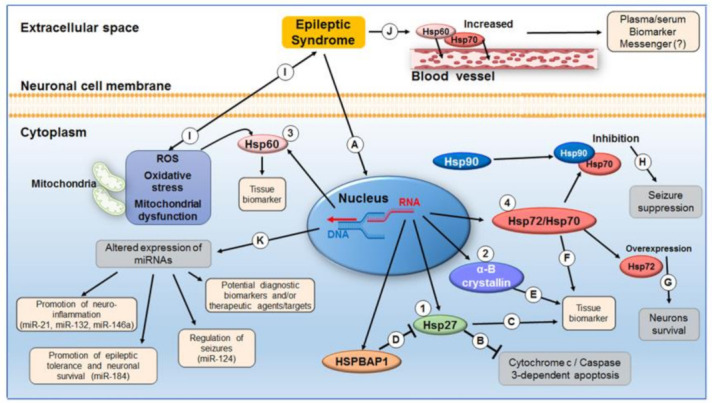
**Molecular chaperones and miRNAs in epilepsy.** The levels of Hsp27 (1), α-B crystallin (2), Hsp60 (3), and Hsp70s (4) are increased in epilepsy (A). Hsp27 could play a cytoprotective role due to its antiapoptotic activity (B) and could be a marker of the cortical tissue affected by seizures (C). The neuroprotective role of Hsp27 is also suggested by the abnormal expression of the Hsp27 inhibitor HSPBAP1 in epileptic tissue (D). In addition, the chaperone α-B crystallin is a promising tissue marker of epileptic foci (E). Likewise, Hsp72 (and other members of the Hsp70 family) increase in the epileptic status and can be considered potential markers of epileptic tissue foci (F). Hsp72 overexpression plays a cytoprotective role by promoting neuronal survival during seizures (G) [47] but overexpression following epileptic stress is accompanied by increased neuronal hyperactivity; thus, pharmacological inhibition of the chaperone suppresses neuronal hyper excitability (H) [48]. A similar seizure-suppression effect was observed following Hsp90-β inhibition (H). Mitochondrial dysfunction, free radical production, and oxidative stress are considered both cause, and consequence of epileptic seizures (I). Mitochondrial Hsp60 increases in response to the oxidative stress induced by epileptic seizures, pointing to its potential as epileptic tissue biomarker [49]. The levels of Hsp70 and Hsp60 increase in plasma and/or serum of epileptic patients following seizures; thus, assessing the levels of these chaperones is a promising way to retrospectively diagnose seizures and may help in patient follow up. sequential measurements after a seizure may reveal profiles useful for predicting a new seizure (J). Altered expression of different miRNAs has an impact on various cellular processes pertinent to epilepsy (K). For instance, miR-21 and miR-132 [39] and miR-146a [35,36] are upregulated following epileptic status both in animal models and MTLE patients showing neuroinflammation; miR-184 is upregulated in KA-treated mice and positively correlates with the development of epileptic tolerance and with neuronal survival following mild and severe seizures [38]; miR-124 is downregulated in patients with epilepsy and rats after drug-induced seizures and, when it is augmented, it suppresses seizures by inhibiting the expression of CREB, a key regulator in epileptogenesis [43].

MicroRNAs (miRNAs) are a class of endogenous 22-nucleotide-long noncoding single stranded RNAs that regulate the expression of target genes post-transcriptionally by affecting either the stability or the translation of their mRNAs [23]. Their biogenesis requires highly regulated cleavage events. The first, intranuclear, involves the Drosha microprocessor that generates a long hairpin-shaped RNA molecule called pre-miRNA. The resulting pre-miRNA is transported via exportin-5 and Ran-GTP to the cytoplasm for processing by the RNAase III enzyme Dicer to form an immature duplex of 20–25 nucleotides [23,24,25,26,27]. Finally, after duplex unwinding by a not-yet-identified helicase-like enzyme, one strand is degraded, whereas the other matures into the miRNA, which subsequently binds to Argonaute (Ago) proteins to form the RNA-induced silencing complex (RISC), able to interact with the specific target mRNAs and inhibit their translation or cause their degradation, depending on whether complementarity to the target mRNA sequence is incomplete or complete, respectively [28]. Over 2000 human miRNAs have been discovered thus far, which regulate the vast majority of protein-encoding genes, and, thus, affect most, if not all, biological events, including cell proliferation, differentiation, and death, playing a significant role during various stages of growth and development [29,30,31,32]. Multiple studies have investigated the variation of miRNA expression patterns in various neurological diseases, including epilepsy, to assess their role in disease pathogenesis and their usefulness as diagnostic biomarkers, as well as to develop efficacious therapeutic strategies [33,34]. Different miRNAs have been implicated in a variety of processes involved in epileptogenesis, such as neuroinflammation, blood brain barrier (BBB) dysfunctions, apoptosis, ion channel dysregulation, axonal guidance, and synaptic plasticity [35,36,37,38,39,40,41,42,43,44], all of which point to them as promising therapeutic targets [45,46] (Figure 1, Table 1).

### MiRNAs and Epilepsy

miRNAs may be involved in the development of epilepsy by regulating those pathological processes mediating epileptogenesis, such as neuroinflammation, neuronal cell death, synaptic remodeling, formation of epileptic circuits, deregulation of neurotrophic factors, and glial cell dysfunction [34]. miRNA expression patterns may be useful for diagnosis and prognostication. For instance, there is a marked variation in miRNA expression patterns in rats with post-status epilepticus, with 19 upregulated and 7 downregulated miRNAs, including miRNA-21, miRNA-22, miRNA-34a, and miRNA-125a, all targeting the mitogen-activated protein kinase (MAPK or MAP kinase) and the long-term potentiation pathways [50]. In plasma of an epileptic rat model, free and exosomal miRNAs, e.g., miR-8071 and the miRNAs associated with the immune response miR-21-5p, miR142-5, and miR-146a-5p, showed different levels compared to healthy controls [51,72]. Exosomal miR-8071 levels were significantly associated with disease duration or seizure frequency [51]. The question of whether miRNAs are altered only during seizures and/or just before and/or after them, or chronically between seizures, has emerged from those observations. The levels of miRNAs in blood can be determined with good reproducibility and are demonstrative of disease status. miRNAs derived from diverse tissues/organs are stable and resistant to nuclease digestion as well as other harsh conditions, including extended storage, freeze–thaw cycles, boiling, and low/high pH [73]. These miRNAs, in biological fluids, may traverse the damaged BBB after epilepsy onset or originate from controlled release in extracellular vesicles (EVs), such as exosomes [74,75].

miRNAs can migrate in exosomes released by CNS cells, including neurons, astrocytes, oligodendrocytes, and microglia [76]. Therefore, it is pertinent to study the role of EVs, including exosomes, in epileptogenic mechanisms and in the establishing of drug resistance, because these mechanisms are still poorly understood [76].

Acute neurological insults and prolonged seizures can regulate miRNA expression in the brain [35,50]. Several miRNAs have been found to be differentially expressed in the hippocampus of TLE or status epilepticus (SE) models. In patients with TLE, a subset of miRNAs was proposed to be a potential regulator of a variety of processes involved in epilepsy, such as neuroinflammation, BBB dysfunctions, apoptosis, ion channel abnormalities, tumors, and disorders of axonal guidance, cell proliferation, neuronal function, and synaptic plasticity [35,36,37,38,39,40,41,42,43,44]. For instance, the inflammation-related miR-21, miR-132 [39], and miR-146a [35,36] are upregulated in chronic stages following epileptic status in animal models and TLE patients. These observations support the notion that neuroinflammation plays a role in the pathogenesis of TLE, with miR-221 and miR-222 targeting the intercellular adhesion molecule 1 (ICAM1, which mediates intercellular interactions in inflammation [77]), being downregulated in MTLE-HS patients [37]. In this case, therapeutic upregulation of miRNAs could reduce neuroinflammation. miR-124 is downregulated in patients with epilepsy and in rats after drug-induced seizures [43], while its upregulation had an antiepileptic effect by inhibiting the expression of CREB, a key regulator in epileptogenesis [43]. miR-184 is upregulated in KA-treated mice and positively correlates with the development of epileptic tolerance and with neuronal survival following mild and severe seizures, while its inhibition increases neuronal death after seizures [38]. On the contrary, miR-134, which has been implicated in the control of dendritic spine morphology [78,79], is upregulated in experimental and human epilepsy [44], while miR-134 silencing with antagomirs generates a seizure-refractory state and attenuates epileptic seizures TLE [44].

Twenty miRNAs with altered expression in the human epileptic hippocampus were identified in MTLE-HS patients, with 19 of them being upregulated and one downregulated [80].

In summary, because the expression of genes depends on the balance of miRNAs present in the cell, an abnormal up or downregulation of specific miRNAs might influence genes and pathways, resulting in pathology. Two strategies for developing miRNA-based therapeutics have been proposed: (a) to develop mimics or agomirs to restore a loss of function of miRNAs and increase their effectiveness; and (b) to produce inhibitors or antagomirs to block endogenous miRNAs and, thus, increase expression of the mRNA targets [81].

## 3. The Chaperone System and the Chaperonopathies

The chaperone (or chaperoning) system (CS) of an organism is composed of molecular chaperones (some of which are named heat shock protein, or Hsp), co-chaperones, chaperone co-factors, and chaperone interactors and receptors [82,83]. The canonical function of the CS is maintenance of protein homeostasis, and, in this, it collaborates with the ubiquitin–proteasome system and with chaperone-mediated autophagy [84,85]. In addition, some components of the CS have non-canonical functions unrelated to protein homeostasis but pertinent to inflammation, autoimmunity, and cancer [86,87,88,89,90,91,92]. Although chaperones are typically cytoprotective, when abnormal, they can become pathogenic and cause diseases, i.e., the chaperonopathies [82,87]. In this part of the article, we discuss critical aspects of epilepsy, in which the canonical and non-canonical functions of the CS are involved, focusing on the chaperones. These have been grouped in various ways; a classification useful to practitioners and to researchers is based on molecular size. The various groups include chaperones within the following molecular weight ranges, in kDa: 200 and over; 100–199; 81–99, 65–80, 55–64, 35–54, and 34 or lower [87,93]. Within these groups are included families of phylogenetically related molecules, such as the Hsp90, Hsp70, and alpha-crystallin chaperones, which belong in the 81–99, 65–80, and 34 or lower ranges (named small Hsp or sHsp), respectively. Within the 55–64 range are the chaperonins, Hsp60, and CCT (chaperonin-containing TCP-1), namely chaperonins of Group I and II, respectively. One or more chaperones in each of those ranges have been implicated in diseases of the nervous system, and we will discuss some that are related to epilepsy.

### 3.1. Molecular Chaperones in Epilepsy

The role of molecular chaperones in epilepsy has not yet been fully clarified. They may be considered markers of an epileptic condition and the associated brain damage/injury, or directly involved in cytoprotective mechanisms in response to seizures [94,95,96,97] (Figure 1, Table 1).

#### 3.1.1. sHsp

Hsp27 (HSPB1; HSP28) belongs to the family of small heat shock proteins, is highly inducible in the CNS in response to various insults, such as hyperthermia, ischemia, hypoxia, and seizures, and contributes to neuroprotection thanks to its active role in protein quality control and its antiapoptotic activity [98]. This cytoprotective activity has also been observed in epileptic brains. Hsp27 was found highly expressed in neocortex obtained from epileptic patients during neurosurgery, with a prevalence in astrocytes and in cerebral blood vessel walls, whereas only low amounts were detected in control brains [52]. Although the reasons for its increase were not elucidated, it was suggested that Hsp27 could serve as marker to specifically localize the cortical regions affected by seizures, which, in turn, could allow the study of the morphological and functional pathology-related alterations. These results and inferences are in agreement with those obtained in rat models, showing a focal increase in Hsp27 after the administration of kainic acid (KA) or pentylenetetrazole (PTZ), both inducing seizures [52,53,54,55], indicating that elevated levels of Hsp27 occurred in a seizure-dependent manner in specific brain areas.

Some evidence indicates that the observed Hsp27 upregulation in specific areas of the brain affected by seizures could be not a mere indicator of brain injury, but also a protective mechanism to counteract the seizure-induced damage. Accordingly, in mice engineered to overexpress human Hsp27, a significantly reduced seizure activity and a decrease in hippocampal damage after the administration of KA was observed [99]. The neuroprotective role of Hsp27 in epilepsy was further confirmed by the abnormal expression of HSPBAP1 (heat shock 27-kDa associated protein 1), observed in the neocortex of patients with intractable epilepsy (IE) [100]. Normally, HSPBAP1 is not expressed in adult brain tissue and, although its functions still remain unclear, it was suggested that they might be opposite to the biological functions of Hsp27 [101]. HSPBAP1 was expressed in the neuronal and glial cells of the temporal lobe of patients with IE, whereas its expression was absent in the control tissues, suggesting its involvement in the pathogenesis and progression of the disease, likely by inhibiting Hsp27 activity. Thus, HSPBAP1 appears an attractive therapeutic target in the treatment of IE patients [100]. However, since the possible functional relationships between Hsp27 and HSPBAP1 have not yet been fully clarified, it cannot be excluded that they can also cooperate in hampering the seizures.

As with Hsp27, α-B crystallin (HSPB5; CRYAD), another molecular chaperone belonging to the small heat shock protein family, was proposed as a promising tissue marker for epileptic foci, enabling the mapping of the extent of the focus and its margins [56]. α-B crystallin was found overexpressed both in astrocytes and oligodendrocytes, including neuron-associated satellite cells and, occasionally, also in neurons of neocortex, hippocampus, and amygdala of brains from epileptic children [56]. Moreover, in some cases, there was a graded immunoreactivity, which was more intense at, or near, the epileptic focus, and, vice versa, diminished and disappeared away from it [56].

#### 3.1.2. Hsp60

Mitochondrial dysfunction, free radical production, and oxidative stress are considered both consequence and cause of epileptic seizures [102]. Studies with animal models have shown that prolonged epileptic seizures result in free radical production; oxidative damage of proteins, lipids, and DNA; and mitochondrial dysfunction, contributing to seizure-induced brain damage [102,103,104,105,106,107,108]. The oxidative stress resulting from mitochondrial dysfunction could be epileptogenic, rendering the brain more susceptible to epileptic seizures and contributing to epilepsy development [102]. These data could explain the observed association between Hsp60 dysfunction and epileptogenesis [57].

**Figure 2:**
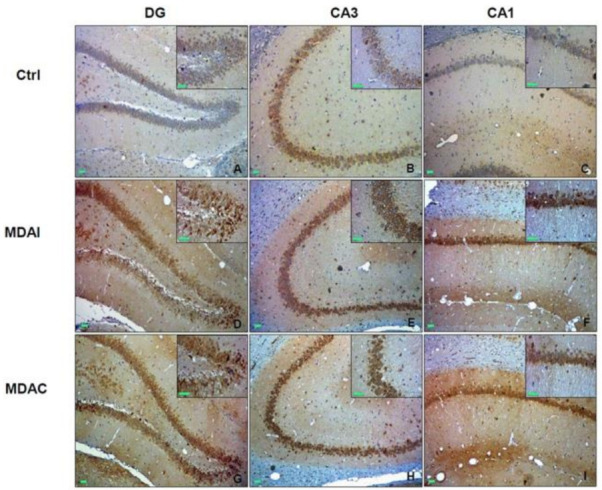
**Hsp60 in experimental temporal lobe epilepsy.** Shown are the levels and preferential localization of Hsp60 in control non-stimulated rats (ctrl) and in maximal dentate activation (MDA)-stimulated rats. (**A**–**C**). Immunohistochemical staining for Hsp60 in the hippocampus of control non-stimulated rats; DG, dentate gyrus; CA3, *Cornu Ammonis* area 3; CA1, *Cornu Ammonis* area 1. (**D**–**F**). Microphotographs of Hsp60 levels in the hippocampus of MDA-stimulated rats, ipsilateral to the perforant pathway stimulation (MDAI). (**G**–**I**). Microphotographs of Hsp60 levels in the hippocampus of MDA-stimulated rats, contralateral to the perforant pathway stimulation (MDAC). As shown in the microphotographs A–C and in the corresponding insets, in ctrl animals, Hsp60 was detected in all the hippocampal sectors. In the DG, Hsp60 immunoreactivity was in the granular cell layer, whereas, in the CA3 and CA1 sectors, it was, for the most part, in the pyramidal cell layer. The images of MDA-stimulated rats (**D**–**I**) and the corresponding insets show that the Hsp60 immunoreactivity was increased in the DG, CA3, and CA1 sectors in MDAI and MDAC compared to ctrl rats. This Hsp60 increase was observed in neuron somata and neuropil. Green scale bar = 100 µm. Reproduced from reference [57], published under the terms and conditions of the Creative Commons Attribution License (CC BY).

Hsp60 (HSPD1; Cpn60) is a group I chaperonin constitutively expressed and further induced under stress conditions. It typically resides within the mitochondrial matrix, where it assists protein folding, ensuring mitochondrial protein homeostasis [109]. However, other non-canonical locations and functions of Hsp60 have been reported [86,110,111]. Hsp60 functional impairment is implicated in mitochondrial dysfunction and oxidative stress, which, in turn, can affect neuronal excitability and seizure susceptibility [49]. Hsp60 levels were found significantly decreased in the hippocampus of epileptic rats, and it was suggested that diminution of the chaperonin could contribute to the development of oxidative stress and increased neuronal excitability [58]. However, Hsp60 levels, as our group previously observed, can also increase in response to the oxidative stress induced by epileptic seizures [57]. For instance, Hsp60 levels were found increased in the dentate gyrus and hippocampus proper and plasma obtained from maximal dentate activation (MDA)-stimulated rats (Figure 2). Likewise, Hsp60 levels in plasma from TLE patients were higher after epileptic seizures than before the seizures and higher than controls, suggesting that this chaperonin can be considered a biomarker of hippocampal stress, potentially useful for diagnosis and patient management [57].

#### 3.1.3. Hsp70

Hsp70 designates a family of chaperones, some members of which are highly expressed in the CNS and believed to be cytoprotective by inhibiting apoptosis [98,112,113,114,115,116,117,118,119]. In neurodegenerative disorders, increased levels of some members of the Hsp70 family are associated with a neuroprotection [120]. The levels of the heat-inducible Hsp70 and its mRNA increased in brains damaged by seizures [59]. In rat models, a direct relationship between frequency, duration, and intensity of seizures induced by KA, flurothyl, or bicuculline administration was observed, and Hsp72 increased in brain regions susceptible to epileptic injury [60,61,62,63]. It remains to be determined whether this Hsp72 augmentation is only a marker for brain injury, or if it has a neuroprotective effect, as suggested by gene transfer experiments [47].

The levels of a non-specified member of the Hsp70 family were investigated in brain tissues from TLE patients and from KA-induced epileptic rodent brains and in primary cultured neurons, and were found increased at the beginning of epileptogenesis, but not at later stages or in dying neurons [64]. Thus, high levels of Hsp70 might be considered a useful indicator for the localization of stressed neurons in the acute phase of epilepsy, but its role in epileptogenesis, protective or otherwise, remains to be elucidated [64]. The reasons for Hsp70 increase were not fully investigated; it could represent a response to stress but without protective effect. In TLE patients, a complete remission of mesial TLE seizures post-surgery was associated with decreased levels of an unspecified Hsp70 in CA4 and subiculum [65]. High Hsp72 levels were observed in the early phase following status epilepticus in a TLE rat model (electrical post-SE model), compared to the later chronic phase, and it was suggested that the chaperone might contribute to the onset of excessive inflammation, triggering molecular and cellular reorganization and generation of a hyperexcitable epileptic network [66]. Thus, the modulation of Hsp72 expression during the early days following an epileptogenic insult could be considered a target for developing antiepileptic therapies. In this regard, it was observed that, in a KA-induced model of TLE, the epileptic stress induced Hsp70 upregulation, resulting in degradation of the Kv4 channels complexes, and, thus, increasing neuronal hyperactivity and exacerbating seizures [48]. Hsp70 pharmacological inhibition suppressed neuronal hyper excitability, attenuating acute or chronic epilepsy by restoring the expression and function of Kv4 channels and, thus, enhancing the A-type current in hippocampal neurons, confirming that the chaperone is a promising therapeutic target for epilepsy treatment [48]. Unfortunately, which member of the Hsp70 family was involved was not stated, limiting the usefulness of this report.

In addition, extracellular Hsps could be useful biomarkers for epileptic conditions. The levels of an unspecified member of the Hsp70 family in serum were significantly higher in epileptic children with febrile seizures than in healthy controls [67]. These results agreed with those from previous studies, also showing that Hsp70 serum levels correlated positively with duration of epilepsy, and negatively with memory scores, hippocampal volume, and cognitive function [68,69,70].

#### 3.1.4. Hsp90

Seizure suppression was observed following Hsp90-β inhibition, which induces an increase in the glutamate transporter-1 (GLT-1) that maintains low extracellular glutamate concentrations [70,71]. Thus, also in this case, chaperone inhibition emerges as a potential antiepileptic strategy.

## 4. Conclusions and Prospects

miRNAs and members of the molecular chaperone system play a central role in the development and maturation of the central and peripheral nervous systems, as well as in their pathology. Thus, the evaluation of variations in their expression patterns has emerged as a promising diagnostic and prognostic parameter, and also as a strategy for designing and implementing novel therapeutic procedures. Epilepsy, which includes a broad spectrum of syndromes, is a serious challenge for clinicians, not only due to the disabling and difficult-to-manage signs, but also due to the elevated frequency of drug-resistant forms, requiring surgical intervention. A novel and promising scenario in the development of new therapeutic options is the identification and modulation of miRNAs that regulate genes involved in processes related to epileptogenesis, such as neuroinflammation, BBB dysfunctions, apoptosis, ion channel dysregulation, axonal guidance, and synaptic plasticity abnormalities. Molecular chaperones, which can be markers of an epileptic condition and the associated brain damage, or directly involved in cytoprotective mechanisms in response to seizures, could help implement targeted therapeutic strategies, aiming to block/inhibit possible detrimental chaperones (negative chaperonotherapy) or, conversely, to promote the activity of the chaperones with a cytoprotective role (positive chaperonotherapy).

## Figures and Tables

**Table 1:** Representative examples of molecular chaperones and miRNAs involved in epileptogenesis.

Molecule		State	Possible Role	Reference
**MiRNAs**	**miR-146a**	Upregulated in chronic stages following epileptic status in TLE patients	Enhancer of neuroinflammation	[35,36]
	**miR-221, miR-222**	Downregulated in MTLE-HS patients	Enhancer of neuroinflammation	[37]
	**miR-184**	Upregulated in KA-treated mice	Promotion of epileptic tolerance and neuronal survival in response to seizures	[38]
	**miR-21, miR-132**	Upregulated in chronic stages following epileptic status in TLE patients	Enhancer of neuroinflammation	[39]
	**miR-124**	Downregulated in patients with epilepsy and in rats after drug-induced seizures	Antiepileptic effect and seizure suppression following miRNA upregulation	[43]
	**miR-134**	Upregulated in experimental and human epilepsy	Enhancer of epileptic seizures and hyperexcitable state	[44]
	**miRNA-21, miRNA-22, miR-34a, and miR-125a**	Decreased in post status epilepticus rat hippocampus and peripheral blood	Possible involvement in molecular mechanisms of neuronal death, inflammation, and epileptogenesis	[50]
	**miR-8071**	Downregulated in exosomes from MTLE-HS patients	Biomarker for disease duration or seizure frequency	[51]
**Molecular chaperones**	**Hsp27**	High levels in neocortex of epileptic patients	Marker to localize the brain regions affected by seizures	[52]
		Focal increased levels in rat brain areas affected by seizures	Marker of epileptic regions	[52,53,54,55]
	**α-B crystallin**	Increased levels in specific brain regions from epileptic children	Tissue marker for epileptic foci	[56]
	**Hsp60**	Increased levels in the dentate gyrus and hippocampus proper and plasma from MDA-stimulated rats	Biomarker of hippocampal stress	[57]
		High levels in plasma from TLE patients	Biomarker of hippocampal stress	[57]
		Decreased levels in the hippocampus of epileptic rats	Induction of oxidative stress and neuronal excitability	[58]
	**Hsp70**	Increased level of heat-inducible Hsp70 in brains damaged by seizures	Neuroprotective	[59]
		Increased level of Hsp72 in rat epileptic brain regions	Marker of brain injury and/or neuroprotection	[47,60,61,62,63]
		High levels at the beginning of epileptogenesis	Indicator for the localization of stressed neurons in the acute phase of epilepsy	[64]
		Decreased levels in CA4 and subiculum from TLE patients post-surgery	Marker of seizure activity	[65]
		High expression of Hsp72 in the early phase following status epilepticus in a TLE rat model	Enhancer of a hyperexcitable epileptic network	[66]
		High levels in KA-induced model of TLE	Enhancer of a hyperexcitable epileptic network through the degradation of the Kv4 channels complexes	[48]
		High levels in serum from epileptic patients	Biomarker for epileptic condition	[67,68,69,70]
	**Hsp90**	Chaperone-induced inhibition	Increase in glutamate transporter-1 (GLT-1) and seizure suppression	[70,71]
